# Primary multiple angiosarcoma of vertebra: A case report

**DOI:** 10.1097/MD.0000000000023587

**Published:** 2020-12-11

**Authors:** Wei Huang, Tao Liu, Ruimeng Duan, Yashuai Yuan, Mingjia Qu, Meng Zhang, Depeng Shang, Xiaobing Yu

**Affiliations:** a Department of Orthopaedics, Zhongshan Hospital of Dalian University, Dalian, 116001, China.; b Department of Spine Surgery, Dongguan Tungwah Hospital, Dongguan, 523000, China.

**Keywords:** angiosarcoma, DNA, High-throughput sequencing, vertebra

## Abstract

**Rationale:**

Angiosarcoma is a rare malignant tumors. The objective of this study is to report a patient who suffered from a progressive low back pain and left lower extremities radiation pain for about 8 months, After diagnoses, this was identified as an extremely rare case of primary multiple angiosarcoma of vertebra.

**Patient concerns:**

A 54-year-old man with a history of 2-year hypertension and 8-year diabetes, both of which were well controlled by drug management. Lately, he suffered from a progressive low back pain and left lower extremities radiation pain for about 8 months.

**Diagnoses:**

Magnetic resonance imaging of lumbar showed a clear pathological fracture and primary multiple angiosarcoma of all vertebra. Postoperative pathology and High-throughput sequencing confirmed the diagnosis of primary multiple angiosarcoma of vertebra.

**Interventions:**

The patient underwent minimally invasive pedicle screw fixation combined with bone cement augmentation for the purpose of stabilizing the damaged vertebrae. Following operation, he received both radiotherapy and chemotherapy for a period of time.

**Outcomes:**

The operation has achieved positive results in relieving pain and stabilizing the spine. No wound problem or operative complications occurred after operation. The patient reported an obvious remission of low back pain and was only capable to perform restricted physiological activities. A long-term palliative radiotherapy and chemotherapy were performed after operation. Unfortunately, the patient died 18 months later.

**Conclusion:**

This article emphasizes primary multiple angiosarcoma of vertebra. Despite being rare, it should be part of the differential when the patient manifested back pain and radiculopathy. We recommended the minimally invasive pedicle screw fixation for angiosarcoma of vertebra. Osteoplasty by bone cement augmentation was also an ideal choice for surgical treatment. It also advocates the use of specific targeted radiotherapy drugs based on gene analysis of tumors.

## 1 Introduction

Angiosarcoma is a rare malignant tumors stemming from vascular or lymphatic endothelial cells, which constitutes approximately 1% to 2% of soft tissue sarcoma. Over 50% of the cases occur in the head and neck, making up 0.1% of the head or neck malignant tumors,^[1]^ for which it has been named angioendothelioma, malignan themangioendothelioma, adenosarcoma, angiosarcoma and lymphangiosarcoma, which are commonly referred to as angiosarcoma.^[1–3]^ With primary or metastatic angiosarcoma of bone (P/MASB) in particular, it is an extremely rare malignant disease, accounting for at maximum 1% of all soft tissue sarcomas.

Tumor tissues are highly differentiated and have a high rate of metastasis and recurrence. Conventional imaging examination is incapable to make accurate diagnosis or provide valuable clues, which makes the process of diagnosis and treatment of angiosarcoma complicated.^[4]^ At present, there remains no standard for treatment. The common practice includes surgical excision, radiotherapy and chemotherapy. However, the effect of chemotherapy has yet to be evaluated.^[5]^ We found a case of vertebral angiosarcoma. We first used percutaneous vertebro plasty to stabilize the vertebral body, and then selected targeted drugs according to the results of DNA sequencing.

## 2 Case report

**Figure 1:**
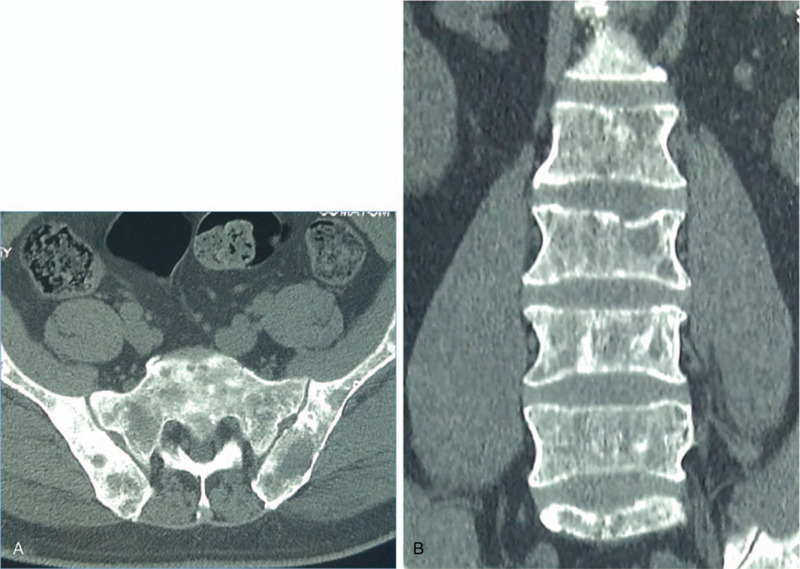
Demonstrating an extensive bone lytic lesion of the whole spine or pelvic bone No tumor in other body soft tissues or organs was discovered by CT (1A for Pelvis CT at horizontal plane, 2A for Lumbar CT at coronal plane).

**Figure 2:**
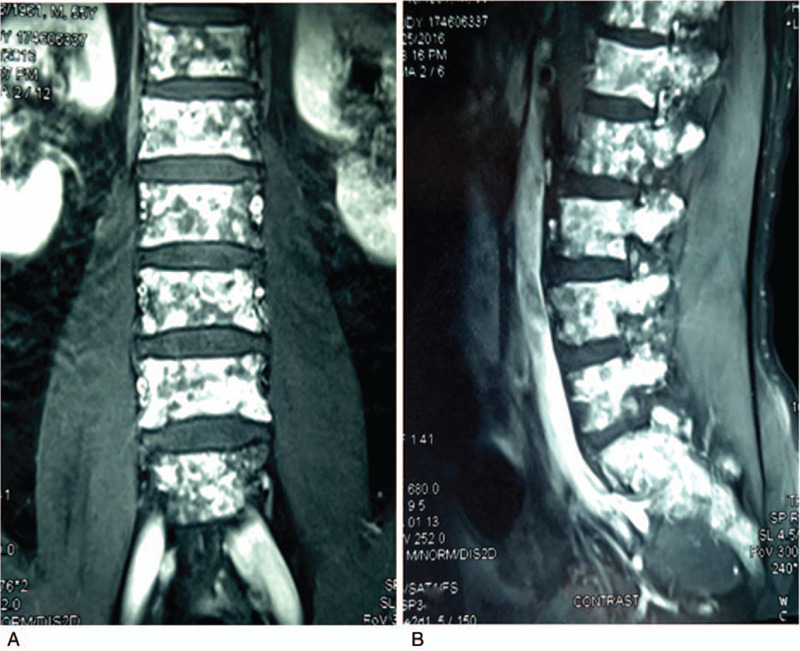
Lumbar MRI exhibiting an extensive bone lytic lesion of the the the whole spine. No tumor in other body soft tissues or organs was identified (2A at coronal plane, 2B at sagittal plane).

In December of 2016, a 54-year-old man with a history of 2-year hypertension and 8-year diabetes, both of which were well controlled by drug management, suffered from a progressive low back pain and left lower extremities radiation pain for about 8 months. The pain in his back could reach 8 points using visual analog scale and could not be alleviated with rest and hot compresses. The imaging examination revealed a multiple spinal pathological fracture with a bone lytic lesion located in the all spinal vertebral body (Figure S1–S5), The shoulder joint consisted of the humerus and clavicle (Figure S6), and pelvis bone (Figs. 1 and 2 and Figure S7–S10). However, no tumor was observed in other tissues and organs except for bone tissue. The blood pressure was measured to be 120/86 mm Hg, the heart rate was measured to be 76/min, and the respiratory rate was measured 16/min.

His blood samples were collected before peripheral blood mononuclear cells DNA was separated. High-throughput mutation sequencing was performed for the full exon regions in 578 cancer related genes. Mutations in NF1 exon 32 (c.4183 C>T p.Gln1395X) and TP53 exon 7 (c.764_766 del, p.255–256 del) were detected, which indicated that the drugs targeting p53 and Nf1, including MEK inhibitor trametinib and cobimetinib, as well as mTOR inhibitor everolimus and temsirolimus had a possibility to be suited to his treatment.

Sequenom MassARRAY system (Sequenom) based on time-of-flight mass spectrometry was performed for the detection of single nucleotide polymorphisms (SNPs) in the genes associated with sensitivity to chemotherapeutic agents. The potential SNPs were detected in multiples genes related to chemotherapeutic sensitivity, including X-Ray Repair Cross Complementing 1 (XRCC1) rs1799782G>A, methylenetetrahydrofolate reductase (MTHFR) rs1801133 AA, P-glycoprotein (ABCB1) rs1045642 GG, rs2032582 A>C, and cytochrome P450 2D6 (CYP2D6) rs1065852 AA. These SNPs were indicative of increased sensitivity to paclitaxel, carboplatin, cisplatin, and oxaliplatin, and reduced sensitivity to tamoxifen, vincristine, and methotrexate.

Preoperative assessments included echocardiogram,electrocardiogram, and chest X-ray. Lumbar magnetic resonance imaging was ordered to visualize the metastatic lesions, to assess the stability of the vertebral column, and to aid in the formulation of a surgical approach. Considering multiple pathological fractures of the spine, minimally invasive pedicle screw fixation combined with percutaneous vertebro plasty was scheduled for him. In brief, posterior circumferential decompression from T7 toT11 internal fixation was performed. Meanwhile, the T7 and L1 vertebral body was injected with bone cement. The visual inspection using the intraoperative fluoroscopy showed the optimal position of all pedicle screws. The incision was closed and the intraoperative blood loss was approximately 400 mL.Thus, erythrocyte 4U was used. Postoperatively, the patient was referred to the regular ward.

**Figure 3:**
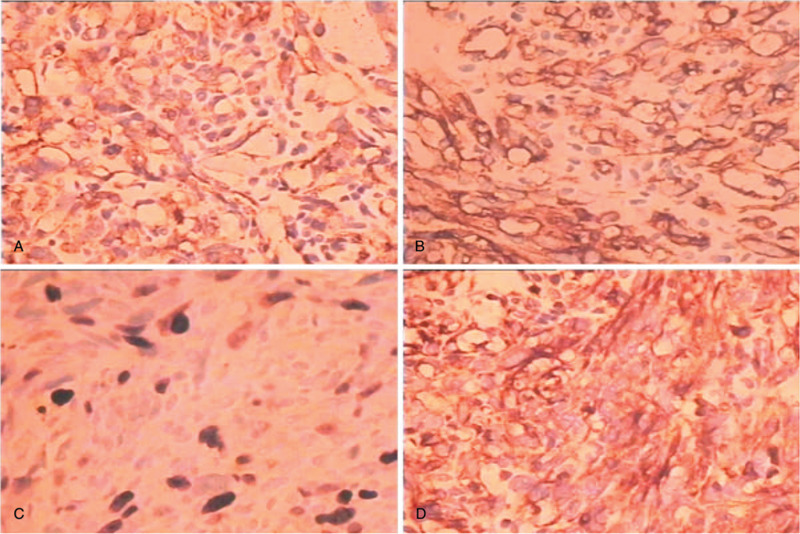
Immunostaining: 3A. CD31 (x400), 3B. CD34(x400), 3C. Ki67 (x400), 4D. Vim(x400), Based on the immunohistochemical findings, the diagnosis was made of a primary angiosarcoma of vertebra.

**Figure 4:**
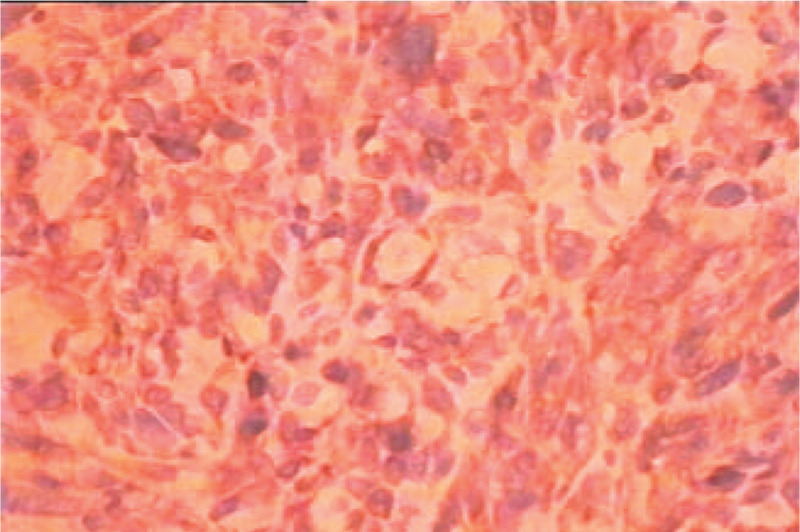
Histopathological section of tumor (H and E stain): Numerous elongated and on occasion branching vascular spaces are highlighted in background of loose connective tissue, hemorrhage and some inflammatory cells. (x400).

**Figure 5:**
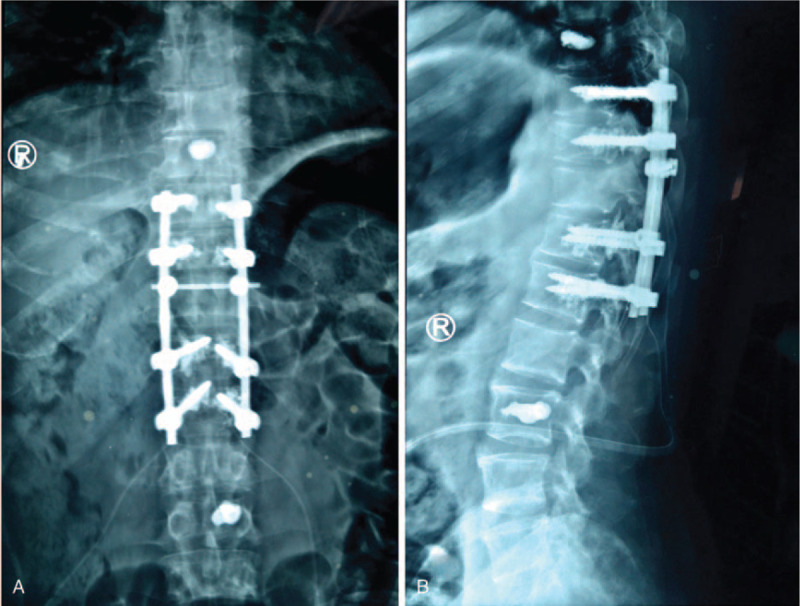
Postoperative X-ray showed that the height of damaged vertebrae was restored and the spinal stability was improved.

The surgical biopsy of the lesion and immunohistochemical examination were conducted to confirm primary angiosarcoma of vertebra (Figs. 3 and 4). Post-operative X-ray confirmed the correct positioning of the implants and there was no sign of displacement for the screws and rods (Fig. 5). The operation achieved positive results in relieving pain and stabilizing the spine. No wound problem or leak of cerebrospinal fluid occurred following the operation.

One week after the operation, the patient's muscle strength of lower extremities improved to grade V compared to the preoperative status, and the symptoms were relieved significantly. Moreover, visual analog scale score of his back pain improved to 0 to 1 points compared to the preoperative status, 7 to 8 points. The patient reported an obvious remission of low back pain and was only capable to perform restricted physiological activities. The palliative radiotherapy and chemotherapy were performed for a long period of time after operation. Unfortunately, the patient died 18 months later.

## 3 Discussion

Primary vascular bone tumors are usually characterized by soluble lesions and sometimes multiple lesions, it is necessary to differentiate between metastatic and multiple myeloma by imaging techniques.^[6]^ In this case, X-ray and CT showed the multiple bone destruction in the patient's body, especially in the spine. We thought it was a multiple myeloma of the lumbar spine at that time, but subsequent pathological biopsy confirmed that the tumor was indeed a primary angiosarcoma. The postoperative pathological tissue sections were characterized by a typical vascular endothelial malignant tumor, where the cells were large cubic and polygonal with eosinophilic cytoplasm and light nucleus. Abnormal mitosis was observed in a large number of cells.^[7]^ It was disorderly and heteromorphic, >2 /10 HPF, immature blood vessels (Fig. 4), and the immunohistochemical staining showed: C K (+ + +), C D 34 (+ + +), C D 31 (+ +). CD31 is a marker of vascular endothelium with high sensitivity and specificity index, it is suggested that the tumors originate from endothelial-like cells.^[8]^ So we diagnosed as epithelioid angiosarcoma of bone (Fig. 3). Angiosarcoma is an aggressive high-grade tumor. The most important differential diagnosis is epithelioid hemangioendothelioma,^[9,10]^ which is a low-grade malignant tumor. It can be easily distinguished that dermoid hemangioendothelioma lacks chondromyxoid matrix and endothelioid cells lack cord-like growth.

Angiosarcoma/hemangiosarcoma in vascular endothelial cells and lymphangiosarcoma stems from lymphatic endothelial cells. There are no reliable morphological and molecular biological indicators to distinguish between the 2, as a result of which they are collectively known as angiosarcoma. It is now believed that the occurrence of angiosarcoma is related to chronic chronic lymphedema and ionization. Radiation, chemical contact, trauma and chronic infection are related to.^[3,4]^ The occurrence of angiosarcoma is associated with the history of trauma and chronic inflammation. As reported by Girard et al.,3 of 28 angiosarcoma cases were associated with a history of trauma at.^[3]^ Naka, and other 99 cases of angiosarcoma led to the discovery that 21% of the patients had a more obvious cause. Of them 5 cases were only inferior to chronic tuberculous pleurisy (6 cases, and the interval of pleurisy and pleural angiosarcoma was 15∼40 years (mean 33 years). Following the analysis conducted of its clinical and pathological characteristics, consideration was given to the long-term inflammatory stimulation. It led to the occurrence of angiosarcoma.

Upon high-throughput sequencing performed for the full exon regions in cancer related genes, a discovery was made of mutations in NF1 exon 32 (c.4183 C>T p.Gln1395X) and TP53 exon 7 (c.764_766 del, p.255–256 del). Accordingly, the therapeutics targeting NF1 MEK inhibitors (trametinib and cobimetinib) and mTOR inhibitors (everolimus and temsirolimus) could be applied to the treatment. Sequenom MassARRAY system analysis was conducted of the SNPs in genes related to the sensitivity to chemotherapeutic agents, with potential SNPs detected in XRCC1 rs1799782G>A, MTHFR rs1801133 AA, ABCB1 rs1045642 GG, rs2032582 A>C, and CYP2D6 rs1065852 AA. These genotypes had been reported to be associated with therapeutic effect or toxicity of tamoxifen, which is a weak anti-estrogen and has a significant inhibitory effect on the expression of CYP2D6 gene.^[11]^ That means the potential of using these therapeutic drugs against this angiosarcom. Previous studies have shown that genotype A/XRCCI Arg399Gin has a higher probability of responding positively to platinum-based therapy in patients with non-small cell lung cancer, suggesting that the SNP mutation leads to DNA repair defects and increased effectiveness of platinum-based chemotherapy. XRCC1 is a scaffold protein involved in repair of single strand breaks after BER. Some studies have shown that XRCC1 Arg399Gln is significantly associated with the prognosis of non-small cell lung cancer. XRCC1 Arg399G/G is significantly associated with better critical survival in patients treated with cisplatin, so cisplatin is also a potential therapy.^[12]^

Generally speaking, the occurrence of angiosarcoma is very rare. Its clinical manifestations are very similar to those of single glomus tumor and Kaposi sarcoma. Without rich clinical experience, it is difficult to diagnose. This requires advanced diagnostic techniques. Pathological biopsy is the most direct and fundamental diagnostic method and the golden standard of diagnosis. Therefore, it is suggested that the patients suspected of hemangiosarcoma should undergo pathological biopsy as soon as possible to determine the type of disease. After definite diagnosis, it is also necessary to detect the gene level of patients. High-throughput sequencing is 1 aspect, to explore the relationship between genes and disease occurrence, and to use specific anticancer drugs to regulate the effect of specific genes. In order to achieve the effect of targeted therapy, and according to the different tissues and organs targeted treatment, for tumors occurring in limbs, it is suggested that radiotherapy and chemotherapy, tumors occurring in bone tissue, should prevent the progress of bone destruction, maintain a certain bone density to play a role in supporting body weight. In a word, the diagnosis and treatment of hemangiosarcoma are very important. Only by definite diagnosis can targeted treatment be carried out.

## Author contributions

**Conceptualization**: Wei Huang, Tao Liu.

**Investigation**: Ruimeng Duan, Depeng Shang.

**Methodology**: Meng Zhang, Yashuai Yuan.

**Resources**: Xiaobing Yu, Meng Zhang.

**Supervision**: Xiaobing Yu, Wei Huang.

**Writing – original draft**: Wei Huang, Mingjia Qu, Xiaobing Yu.

**Writing – review & editing**: Wei Huang, Xiaobing Yu.
